# STAT3 is constitutively acetylated on lysine 685 residues in chronic lymphocytic leukemia cells

**DOI:** 10.18632/oncotarget.26110

**Published:** 2018-09-14

**Authors:** Uri Rozovski, David M. Harris, Ping Li, Zhiming Liu, Preetesh Jain, Alessandra Ferrajoli, Jan Burger, Phillip Thompson, Nitin Jain, William Wierda, Michael J. Keating, Zeev Estrov

**Affiliations:** ^1^ Department of Leukemia, The University of Texas MD Anderson Cancer Center, Houston, Texas, USA; ^2^ Institute of Hematology, Davidoff Cancer Center, Rabin Medical Center, Sackler School of Medicine, Tel Aviv University, Tel Aviv, Israel

**Keywords:** CLL, acetylation, STAT3

## Abstract

Signal transducer and activator of transcription (STAT)-3 might be phosphorylated or acetylated. Unlike the phosphorylation of STAT3, little is known about the acetylation of STAT3 in chronic lymphocytic leukemia (CLL) cells. Because acetylation activates STAT3 transcription, we sought to study the acetylation status of STAT3 in CLL cells. Using Western immunoblotting, immunoprecipitation, and flow cytometry we found that, apart from its constitutive serine phosphorylation, STAT3 is constitutively acetylated on lysine 685 residues. Because the acetyltransferase p300 was found to acetylate STAT3 on lysine 685 residues, we wondered whether p300 acetylates STAT3 in CLL cells. Using Western immunoblotting we detected high levels of p300 protein in CLL but not normal B cells. Transfection of CLL cells with p300 small-interfering (si) RNA downregulated p300 transcripts as well as p300 and acetyl-STAT3 protein levels. In addition, p300 siRNA attenuated STAT3–DNA binding and downregulated mRNA levels of STAT3-regulated genes. Furthermore, transfection of CLL cells with p300-siRNA induced a 3-fold increase in the rate of spontaneous apoptosis. Taken together, our data suggest that in CLL cells STAT3 p300 induces constitutive acetylation and activation of STAT3. Whether inhibition of STAT3 acetylation might provide clinical benefit in patients with CLL remains to be determined.

## INTRODUCTION

Chronic lymphocytic leukemia (CLL), the most common adult leukemia in the Western hemisphere, is characterized by gradual accumulation of mature appearing CD5+ B lymphocytes [1]. Several transcription factors, including nuclear factor kappa-light-chain-enhancer of activated B cells (NF-κB), nuclear factor of activated T cells (NFAT) and signal transducer and activator of transcription-3 (STAT3), provide CLL cells with a prolonged life span [2–6].

Like NF-κB and NFAT, STAT3 activates a pleiotropic cascade which ultimately induces the expression of pro-survival and anti-apoptotic genes [7]. In most eukaryotic cells STAT3 is ubiquitously expressed [8] and conditionally activated by post-translational modifications such as phosphorylation, methylation and acetylation [3, 9–11]. Post-translational activation of STAT3 is usually induced by extracellular stimuli that activate signal transduction and transcription. However, unlike normal cells, several neoplastic cells do not require an extracellular stimulus to initiate transcription. In CLL cells STAT3 is constitutively phosphorylated (p) on serine residues and serine phosphorylated STAT3 (pSTAT3) forms homodimers and heterodimers, shuttles to the nucleus and induces the expression of STAT3-target genes [6]. Whereas several investigators studied the induction and effects of pSTAT3, little is known about the acetylation of STAT3 in CLL cells. Because acetylation activates the transcriptional activity of STAT3 [10] and the acetyltransferase p300 acetylates STAT3 on lysine 685 residues [12], we sought to determine whether p300 acetylates STAT3 and whether acetylation contributes to the transcriptional activity of STAT3 in CLL cells.

## RESULTS

### STAT3 is constitutively acetylated on lysine 685 residues in CLL cells

**Figure 1:**
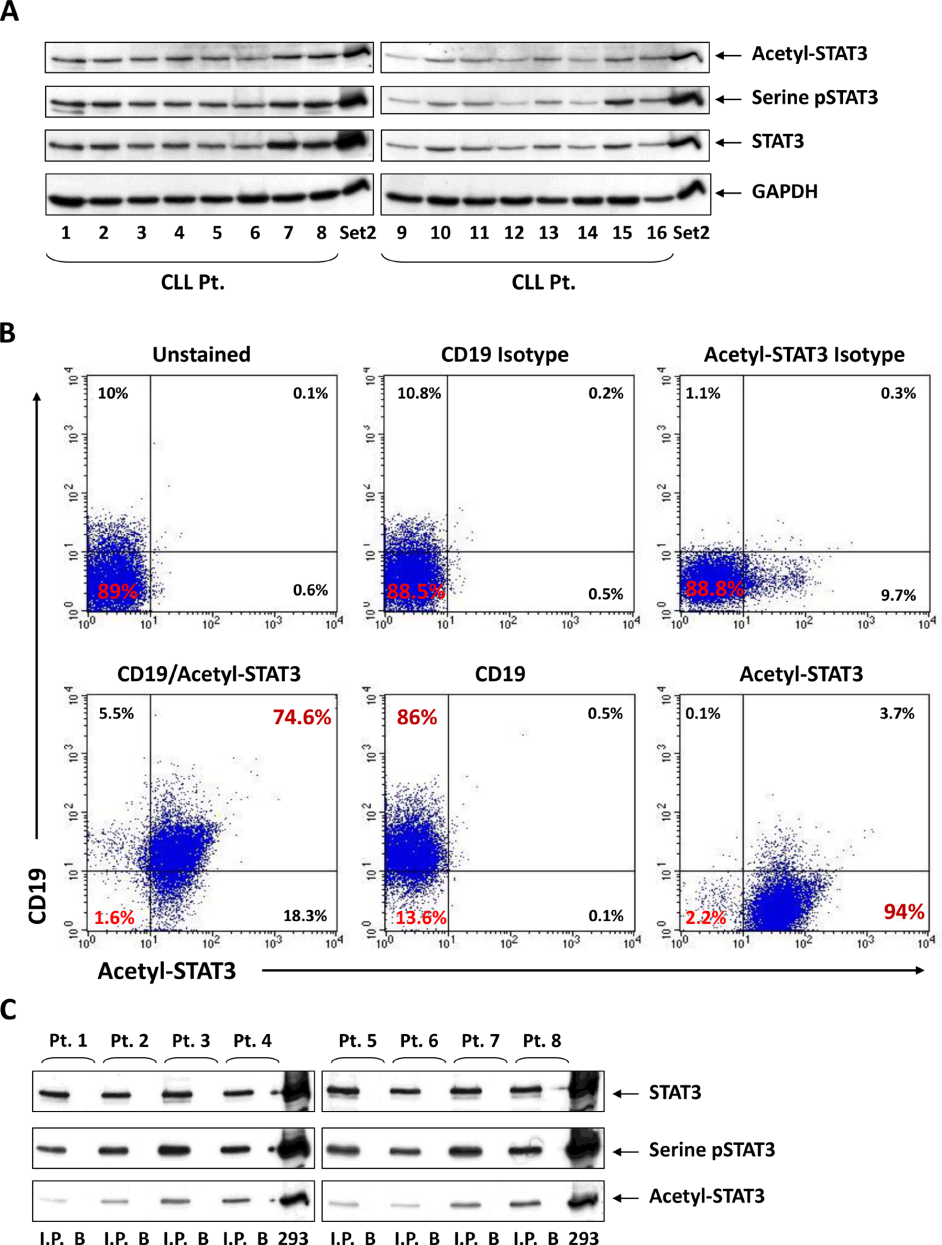
STAT3 is constitutively acetylated on lysine 685 residues in CLL cells (**A**) Cell lysates of cells from 16 patients with CLL were analyzed by Western immunoblotting using acetyl-STAT3, serine pSTAT3 and total STAT3 antibodies. Set2 cells were used as positive controls and GAPDH as loading control. (**B**) Low density PB cells were double stained for CD19 and acetyl-STAT3 antibodies and their corresponding isotype antibodies. In CLL cells of this patient, 74.6% of the cells stained positively for both CD19 and acetyl-STAT3. A representative experiment from 5 experiments that were conducted using PB samples from 5 different patients is depicted. (**C**) Acetyl-STAT3 and serine pSTAT3 co-immunoprecipitated with STAT3 antibodies. CLL cell lysates from 8 patients were immunoprecipitated with anti-STAT3 antibodies. The immune complex was then separated using SDS-PAGE. In all patient samples, serine pSTAT3 and acetyl-STAT3 were readily detected. HEK 293 cells were used as a positive control. Total STAT3 antibodies were used as loading control. I.P., Immunoprecipitate; B, beads coated with STAT3 isotype antibodies.

To test whether STAT3 is acetylated on lysine residues in CLL cells we isolated PB low-density cells from 16 randomly selected, untreated CLL patients and performed Western immunoblotting. We found that STAT3 was constitutively acetylated on lysine 685 residues in CLL cells from all 16 patients, regardless of the patients’ clinical characteristics, cytogenetic abnormalities or IgHV mutation status (Supplementary Table 1). In agreement with our previous observation [6], we also found that in all patient samples that STAT3 was constitutively phosphorylated on serine 727 residues (Figure 1A). Flow cytometry analysis of 5 CLL patients revealed that 55% to 75% of the PB low-density cells co-expressed CD19 and acetyl-STAT3 (Figure 1B). To confirm and further delineate these findings, we immunoprecipitated cell protein obtained from PB low-density cells of 8 randomly selected CLL patients with anti-STAT3 antibodies and, as shown in Figure 1C, we detected serine pSTAT3 and acetyl-STAT3 in the immuneprecipitate.

### Acetylation and phosphorylation of STAT3 are independent events

**Figure 2:**
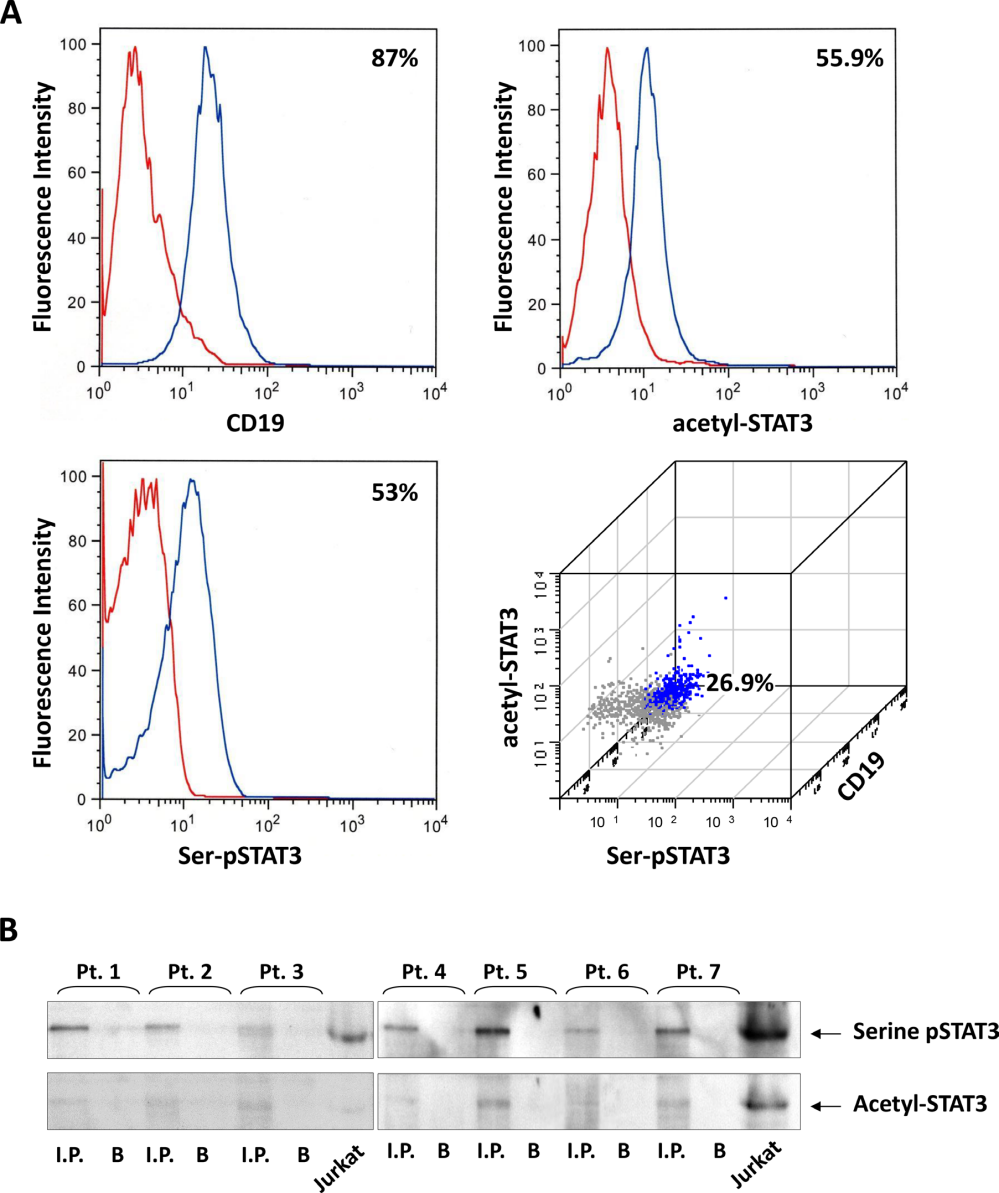
STAT3 is constitutively acetylated on Lysine 685 residues (**A**) Low density PB cells from 4 patients were stained with CD19, serine pSTAT3 and acetyl-STAT3 antibodies and analyzed using flow cytometry. The rate of cells expressing CD19 was 87%, of serine pSTAT3 was 53%, of cells expressing acetyl-STAT3 was 55.9%, and of cells expressing CD19, serine pSTAT3 and acetyl-STAT3 was 26.9%. (**B**) CLL cell protein from PB samples of 7 patients was immunoprecipitated with anti-serine pSTAT3 antbodies. As shown, acetyl-STAT3 was detected in the serine pSTAT3 immunoprecipitate of all patients. Jurkat cells were used as a positive control. I.P., Immunoprecipitate; B, beads coated with serine pSTAT3 isotype antibodies.

Because serine pSTAT3 and acetyl STAT3 co-immunoprecipitated with anti-STAT3 antibodies (Figure 1C), we wondered whether STAT3 phosphorylation and acetylation are concomitant or independent events. Using flow cytometry, we quantified the rates of either STAT3 serine phosphorylation, lysine acetylation or both in CLL cells from 4 different patients. As shown in Figure 2A, the rate of cells co-expressing CD19, acetyl-STAT3 and serine pSTAT3 was 27% whereas the rate of acetyl-STAT3 was 56% and of serine pSTAT3 was 53%, suggesting that phosphorylation and acetylation of STAT3 are independent events. To determine whether in a subpopulation of CLL cells STAT3 is both phosphorylated and acetylated, we immunoprecipitated CLL-cell protein with anti-serine pSTAT3 antibodies and found that acetyl-STAT3 was readily detected in the serine pSTAT3 immunoprecipitate (Figure 2B), suggesting that in a subpopulation of CLL cells STAT3 is both serine-phosphorylated and lysine-acetylated.

### p300 acetylates STAT3 in CLL cells

**Figure 3:**
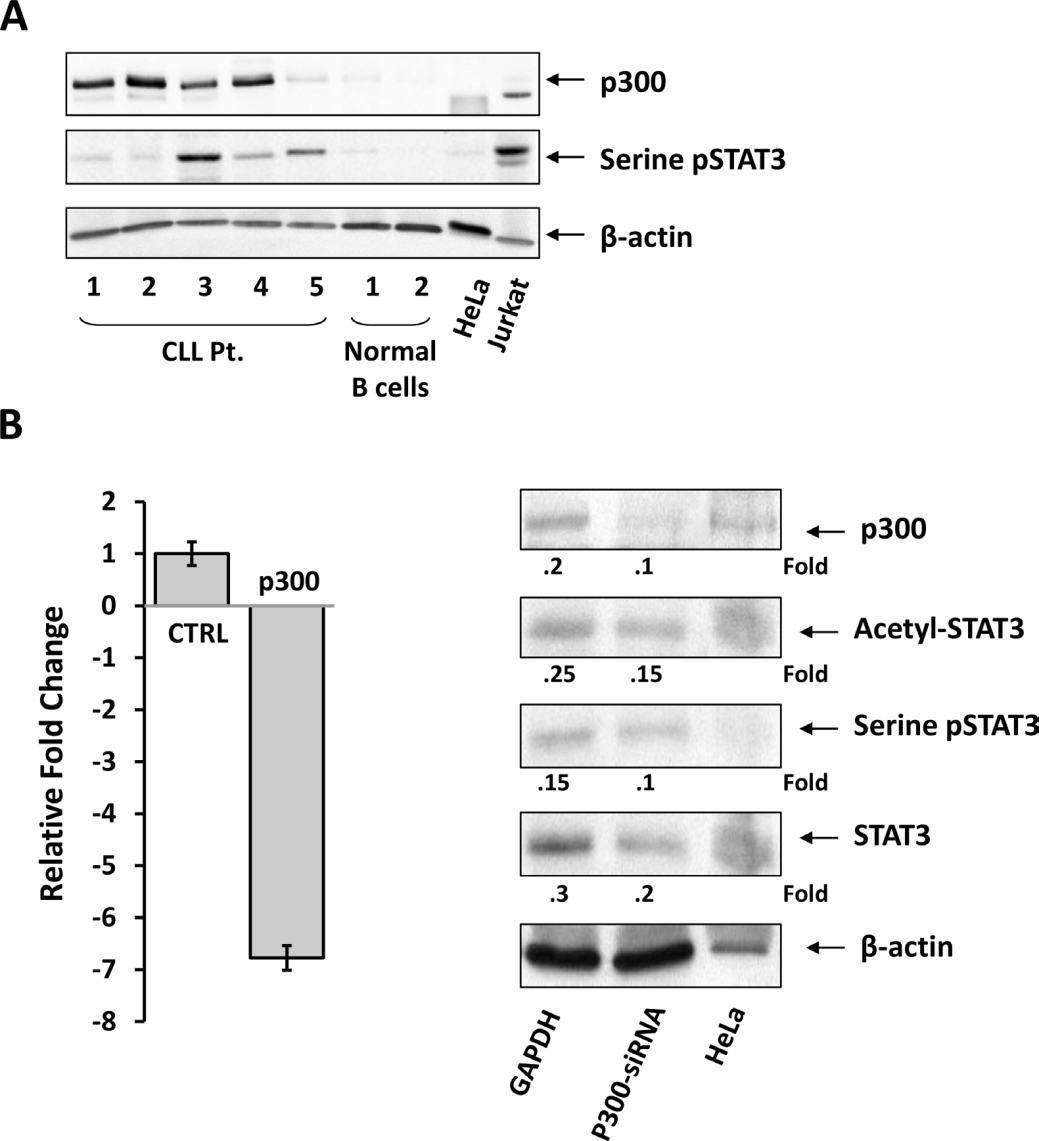
p300 acetylates STAT3 in CLL cells (**A**) Western immunoblotting showing increased levels of p300 in 4 patients and low levels in one patient with CLL but not in CD19+ B cells from 2 healthy volunteers. HELA and Jurkat cells were used as controls. (**B**) CLL cells were transfected with p300-siRNA or GAPDH using electroporation. As shown in the left panel, qRT-PCR analysis showed that transfection with p300-siRNA downregulated p300 transcript levels by approximately 7-fold. A Western blot analysis depicted in the right panel showed that transfection of CLL cells with p300-siRNA downregulated p300, acetyl-STAT3, serine pSTAT3, and STAT3 protein levels. A representative of 4 experiment using samples of 4 different patients yielded similar results. A representative experiment is depicted.

Because STAT3 is constitutively acetylated in CLL cells (Figure 1A) and p300 was found to acetylate STAT3 in other cell types [13], we wondered whether p300 also acetylates STAT3 in CLL cells. To answer this question, we performed Western immunoblotting using PB low-density cells from 5 CLL patients and CD19+ B cells from of 2 healthy donors. Our results showed that, like serine pSTAT3, p300 was detected at high levels in CLL but not in normal B cells (Figure 3A). To determine whether p300 induces acetylation of STAT3 in CLL cells, we transfected CLL cells obtained from 4 different patients with p300-siRNA. At a transfection efficiency of approximately 30%, p300-siRNA downregulated p300 transcript levels, p300, acetyl-STAT3, serine pSTAT3, and STAT3 protein levels (Figure 3B; Supplementary Figure 1).

### Acetyl-STAT3 activates STAT3 transcription and provides CLL cells with a survival advantage

**Figure 4:**
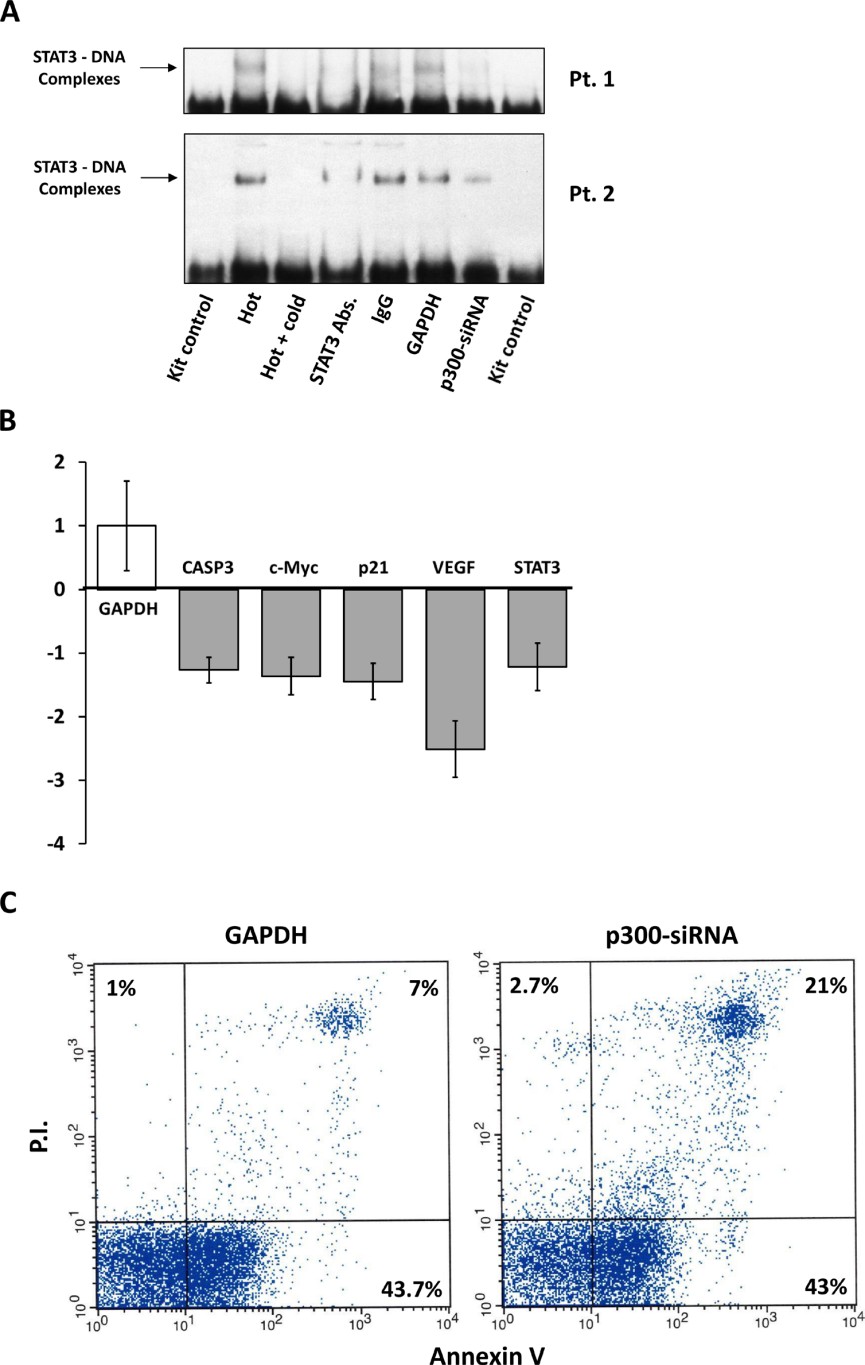
Acetylation of STAT3 activates STAT3 transcription and provides CLL cells with survival advantage (**A**) Nuclear extracts of untransfected or p300-siRNA-transfected CLL cells from 2 patients were incubated with biotinylated DNA harboring STAT3 binding sites. EMSA showed that the addition of excess unlabeled probe, anti-STAT3 antibodies, but not their isotype IgG, or transfection with p300-siRNA, but not with GAPDH, attenuated the binding of the cell extract to the labeled DNA probe, suggesting that transfection with p300-siRNA inhibits STAT3-DNA binding. (**B**) CLL cells were transfected with p300-siRNA or with GAPDH and qRT-PCR was used to determine the levels of STAT3-regulated genes. As shown, levels of *CASP3, c-Myc, P21, VEGF,* and *STAT3* mRNA levels were downregulated in p300-siRNA-transfected cells. (**C**) Flow cytometry analysis of CLL cells transfected with p300-siRNA or with GAPDH. Compared with GAPDH-transfected cells the rate of active apoptosis (Annexin/PI positive) were 3 folds higher in p300-siRNA transfected cells.

To test whether STAT3 acetylation increases transcriptional activity of STAT3 we first assessed whether acetylation of STAT3 contributes to STAT3-DNA binding. Using EMSA we found that transfection of CLL cells with p300-siRNA attenuated STAT3–DNA binding (Figure 4A). Furthermore, we found that transfection of CLL cells with p300-siRNA downregulated expression levels of *Caspase3, c-Myc*, *p21, VEGF*, and *STAT3* transcript levels (Figure 4B), confirming that acetylation increases the transcriptional activity of STAT3 in CLL cells.

Because STAT3 activates anti-apoptotic pathways [3, 6, 14, 15] and p300 induced the acetylation and activation of STAT3, we wondered whether transfection of CLL cells with p300-siRNA would affect the spontaneous apoptosis rate of CLL cells. We found that transfection of CLL cells with p300-siRNA induced a 3-fold increase in the rate of spontaneous apoptosis compared to rate of spontaneous apoptosis in cells transfected with GAPDH, suggesting that p300-induced acetylation of STAT3 provides CLL with survival advantage (Figure 4C).

## DISCUSSION

Here we show that in CLL cells STAT3 is constitutively acetylated on lysine 685 residues and that acetyl-STAT3 provides CLL cells with a survival advantage.

Accumulating data suggest that, similar to other post-translational modifications, acetylation affects both epigenetic regulation and signal transduction [16]. Inducible STAT3 acetylation occurs during inflammation [11, 17] at which time acetylated STAT3 activates pro-survival pathways in a variety of human cancer cells [18] by stabilizing STAT3-STAT3 dimers [10, 19], increasing DNA binding affinity [10, 12], enhancing transcriptional activation [10, 12, 20], and promoting protein-protein interactions [10, 12, 19, 20].

Acetylation is typically described as a highly reversible process [21]. However, our data suggest that in CLL cells STAT3 is constitutively acetylated on lysine 685 residues, likely because CLL cells harbor high levels of p300 that acetylates STAT3. We found that in approximately 50% of PB CLL cells STAT3 is constitutively acetylated, a rate which is similar to that of constitutive serine pSTAT3. Furthermore, serine phosphorylation and lysine acetylation appear to be independently in most, and concomitant in a small fraction of CLL cells. In a previous study we have shown that STAT3 undergoes tyrosine phosphorylation following stimulation of the B-cell receptor or in response to IL-6 or activation [3]. Together, these post-transcriptional modifications represent several converging independent events leading to activation of STAT3. Each protein modification leads to an increase in STAT3-DNA binding and promotes the transcription of a plethora of STAT3 target genes.

Although overtly effective *in vitro*, inhibitors of tyrosine pSTAT3 or lysine acetyl- STAT3 were found to possess limited clinical activity [22]. Because multiple alternative post-translational pathways activate STAT3, inhibiting translation might prove to be more effective. Recently, this approach yielded encouraging results as STAT3 antisense oligonucleotides induced clinical responses in patients with lymphoma and lung cancer [23].

Aberrant p300 was found to affect non-histone substrates and facilitate the tumorigenic potential of several oncogenes including NOTCH3, AML1 and cMyb [24]. Similar to those findings, our data suggest that p300, present at relatively high levels in CLL cells, induces acetylation of STAT3 and that acetylated STAT3 provides CLL cells with survival advantage. Whether p300 acetylates other proteins in CLL cells and whether inhibiting its activity might provide clinical benefits to patients with CLL remain to be determined.

## MATERIALS AND METHODS

### Patients’ characteristics

Peripheral blood samples were obtained from randomly chosen 40 patients with CLL who were treated at The University of Texas MD Anderson Cancer Center Clinic. The study was approved by our Institutional Review Board and patients’ Informed Consent was obtained prior to sample collection. Clinical characteristics of all patients that participated in this study are depicted in Supplementary Table 1.

### Cell fractionation

PB cells were fractionated using Ficoll Hypaque 1077 (Sigma, St. Louis, MO, USA). The low-density cellular fraction was used immediately or frozen for additional studies. More than 95% of the peripheral blood lymphocytes obtained from these patients were CD19+/CD5+, as assessed by flow cytometry (Becton, Dickinson and Company, Franklin Lakes, NJ, USA). As control studies we obtained from the Central Blood Bank left-over buffy coats of healthy blood donors. After Ficoll-Hypaque fractionation, the donors’ B cells were isolated using Miltenyi CD19-coated beads according to the manufacturer's instructions (Miltenyi Biotec, Bergisch Gladbach, Germany).

### Western blot analysis

Western blot analysis was performed as previously described [25]. Briefly, cell lysates were assayed for their protein concentrations using the BCA protein assay reagent (Pierce Chemical). Each set of paired lysates was adjusted to the same protein concentration. Lysates of CLL cell extract were mixed with 4× Laemmli sample buffer and was then denatured by boiling for 5 minutes. Forty micrograms of lysates were separated using 8% sodium dodecyl sulfate–polyacrylamide gel electrophoresis and then transferred to a nitrocellulose. The transfer was done overnight at 30 V in a cooled (4° C) reservoir. The nitrocellulose membrane was then placed in a Ponceaus S solution to verify equal loading of protein. The membranes were blocked with 5% dried milk dissolved in 50 mL of phosphate-buffered saline (PBS). After blocking, the membrane was incubated with the following primary antibodies: monoclonal mouse anti-human STAT3 (BD Bioscience, Palo Alto, CA, USA), monoclonal mouse anti-human Ser 727 STAT3 (Cell Signaling, Danvers, MA, USA), Rabbit anti-human acetyl STAT3 (Lys 685) antibodies (Cell Signaling), mouse anti-human p300 antibodies (Pierce Biotechnology Inc./Thermo scientific, Rockville, IL), and mouse anti-human β-actin (Sigma-Aldrich, St. Louis, MO, USA). After incubation with horseradish peroxidase–conjugated secondary antibodies (GE Healthcare, Buckinghamshire, UK) for 1 hour, blots were visualized with an enhanced chemiluminescence detection system (GE Healthcare).

### Flow cytometry

CLL cells were fixed in 2% paraformaldehyde for 10 minutes at 37° C and permeabilized overnight at –20° C. Before staining cells were washed three times in PBS with 2% FBS. Cells were then stained with CD19 (BD Biosciences, San Jose, CA, USA), serine phopho-STAT3 (BD Biosciences), and acetyl-STAT3 (Cell Signaling, Beverly, MA, USA) as well as appropriate isotypic controls. Cells were analyzed on a FacsCaliber flow cytometer (BD Biosciences) and data analysis performed using CellQuest software (BD Biosciences). Graphics were created with CellQuest (BD Biosciences) and WinList (Verity Software House, Topsham, ME, USA) software.

### Immunoprecipitation

Immunoprecipitation studies were done as previously described [26]. Briefly, CLL cell lysates were incubated with either STAT3 or with anti–serine pSTAT3 antibodies for 16 hours at 4° C. Protein A agarose beads (EMD Millipore) were added for 2 hours at 4° C. For negative controls, the lysates were incubated with their corresponding isotype antibodies plus protein A agarose beads. After three washes with immunoprecipitation assay buffer, the beads were suspended in sodium dodecyl sulfate (SDS) sample buffer, boiled for 5 minutes, and removed by centrifugation; and the supernatant proteins were separated by SDS–polyacrylamide gel electrophoresis (PAGE). Human Embryonic Kidney 293 (HEK293) or immortalized human T cell lines were used as positive controls in the immunoprecipitation studies.

### Transfection of CLL cells with p300 small interfering RNA (siRNA)

Five microliters of siPORT NeoFX agent and 50 pmol of either siRNA targeting p300 (Thermo Fisher Life Technologies, Carlsbad, CA, USA) or the FAM-labeled siRNA targeting the human glyceraldehyde 3-phosphate dehydrogenase (GAPDH; Thermo Fisher Life Technologies, Carlsbad, CA, USA) were each diluted in 50 μL of OPTI-MEM I and then mixed together and incubated at room temperature for 10 minutes. A total of 5 × 10^6^ cells suspended in 0.2 mL of OPTI-MEM I medium containing the siRNA and transfection agent was incubated at room temperature. After 1 hour of incubation, transfections were performed by electroporation (Bio-Rad Laboratories) and the cells were cultured in complete RPMI 1640 medium. Transfection efficiency was calculated on the basis of the fluorescent Fluorescein isothiocyanate (FAM)-conjugated siRNA measured by flow cytometry (BD Biosciences).

### RNA extraction

RNA was purified using an RNeasy purification procedure (QIAGEN). RNA quality and concentration were analyzed with a spectrophotometer (ND-1000; NanoDrop Technologies).

### Quantitative reverse-transcription polymerase chain reaction analysis (qRT-PCR)

A total of 500 ng RNA was used in one-step qRT-PCR (Applied Biosystems) with an ABI PRISM 7700 sequence detection system (Applied Biosystems) using a TaqMan gene expression assay for *cMyc, STAT3, p21, CASP3* and *VEGF* in accordance with the manufacturer's instructions. Samples were run in triplicate, and relative quantification was performed by using the comparative C_T_ method.

### Electrophoretic mobility shift assay

Non-denatured cellular nuclear extracts were prepared using a NE-PER extraction kit (Thermo Scientific Pierce, Rockford, IL, USA). Nuclear protein extracts were incubated with biotin-labeled STAT3 DNA probes (Integrated DNA Technologies, San Diego, CA, USA) in binding buffer for 30 minutes on ice. Following incubation, the samples were separated on a 5% polyacrylamide gel, transferred onto a nylon membrane, and fixed on the membrane via ultraviolet cross-linking. The biotin-labeled probe was detected with streptavidin-horseradish peroxidase (Gel-Shift Kit; Panomics, Fremont, CA, USA). The control consisted of 7-fold excess unlabeled cold probe.

### Annexin V/propidium iodide assay

The rate of cellular apoptosis was analyzed using double staining with a Cy5-conjugated annexin V kit and propidium iodide (PI; BD Biosciences) according to the manufacturer's instructions. Briefly, 1 × 10^6^ cells were washed once with phosphate-buffered saline and resuspended in 200 μL binding buffer with 0.5 μg/mL annexin V-Cy5 and 2 μg/ml propidium iodide (PI). After incubation for 15 minutes in the dark at room temperature, the samples were analyzed on a FACSCalibur flow cytometer (BD Biosciences). Cell viability was calculated as the percentage of annexin V-negative cells.

## SUPPLEMENTARY MATERIALS FIGURES AND TABLES


