# Supplementary material for: STAT3 is constitutively acetylated on lysine 685 residues in chronic lymphocytic leukemia cells
Source: Oncotarget. 2018 Sep 14;9(72):33710–8. doi: 10.18632/oncotarget.26110 (PMC6154750; doi:10.18632/oncotarget.26110)
Supplement: Supplementary file 1 [file oncotarget-09-33710-s001.pdf]

STAT3 is constitutively acetylated on lysine 685 residues in chronic lymphocytic leukemia cells

SUPPLEMENTARY MATERIALS

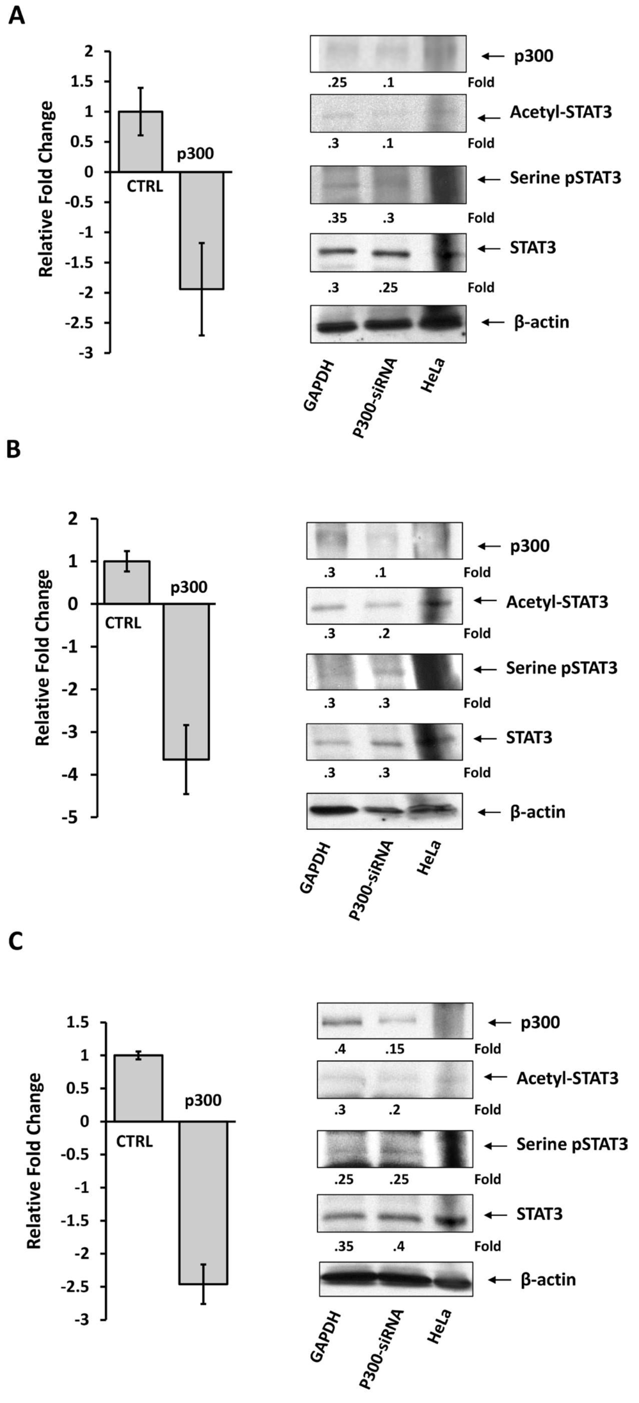

Supplementary Figure 1: CLL cells from 3 (**A**–**C**) different patients were transfected with p300-siRNA or GAPDH using electroporation. As shown in the left panels, qRT-PCR analysis showed that transfection with p300-siRNA downregulated p300 transcript levels. Western blot analyses depicted in the right panels showed that transfection of CLL cells with p300-siRNA downregulated p300, acetyl-STAT3.

Supplementary Table 1: Baseline patient characteristics (*n* = 40)

Characteristic	Measure/Category	Overall
**Age, years**	Median (range)	61 (36–86)
**WBC** $\times 10^9$/L	Median (range)	23 (7–163)
**ALC** $\times 10^9$/L	Median (range)	16 (2–143)
**Rai stage**	(0, 1–2/3–4) (%)	(90/10)
**CD38**	≤30/>30 (%)	77/23 (%)
**Zap-70**	Negative/Positive	80/20 (%)
**β2M (mg/L)**	(</≥ 4 mg/L)	92/8 (%)
**IGHV mutation**	(M/UM)	66/34 (%)
**FISH result, *n***	del17p/11q/T12/13q/Negative	3/3/5/19/10
**Karyotype, *n***	Diploid/Non-diploid/Complex/not done	28/6/2/4
**Survival status, (*n*)**	Alive/Dead	39/1

WBC, white blood cell count; ALC, absolute lymphocyte count; ×2M, beta-2 microglobulin; M, mutated; UM, unmutated; FISH, fluorescence *in situ* hybridization.
